# Long-Term Effects of Intensive Lifestyle Intervention on Nursing Home Stays and Institutional Days

**DOI:** 10.1093/geroni/igaf122.468

**Published:** 2025-12-31

**Authors:** Peter Huckfeldt, Ann Harada, Lynne Wagenknecht, Charles Semelka, Mark Woodhouse, Govinda Yost, Mark Espeland, Denise Houston

**Affiliations:** University of Minnesota, Minneapolis, Minnesota, United States; Leonard D. Schaeffer Center for Health Policy & Economics, Los Angeles, California, United States; Wake Forest University School of Medicine, Winston-Salem, North Carolina, United States; Wake Forest University School of Medicine, Winston-Salem, North Carolina, United States; University of Minnesota, Minneapolis, Minnesota, United States; University of Minnesota School Of Public Health, Minneapolis, Minnesota, United States; Wake Forest University School of Medicine, Winston-Salem, North Carolina, United States; Wake Forest University School of Medicine, Winston-Salem, North Carolina, United States

## Abstract

Diabetes and obesity can accelerate aging processes and functional decline and are associated with a higher lifetime risk of nursing home (NH) admission among older adults. The Look AHEAD study randomized 5,145 participants with type 2 diabetes and overweight or obesity to an intensive lifestyle intervention (ILI) focused on weight loss and increased physical activity compared to a control group receiving diabetes support and education. The Look AHEAD ILI led to long-term improvements in mobility, physical function, and moderate/severe physical disability. These benefits could translate to lower needs for care received in a NH, hospital, or post-acute care facility. We linked 3038 consenting participants in the Look AHEAD study to Medicare data and identified short NH stays (90 or fewer days) and long NH stays (more than 90 days) in the Long Term Care Minimum Data Set in the eight years immediately following the termination of the ∼10-year intervention (2013-2020). We found that 22.2% of ILI and 22.1% of controls received any NH care during this period and 1.9% of ILI and 2.9% of controls had any long NH stays. There was no significant difference between arms (p = 0.706 for any NH and p = 0.093 for long NH stays), suggesting that improvements in functional status did not translate to reduced NH admissions. In ongoing work, we are examining the association of improved mobility and physical function during and after the intervention with “healthy days at home”, which are defined as days not spent in a hospital, post-acute care facility, or NH.

